# Short Insertion and Deletion Discoveries via Whole-Genome Sequencing of 101 Thoroughbred Racehorses

**DOI:** 10.3390/genes14030638

**Published:** 2023-03-03

**Authors:** Teruaki Tozaki, Aoi Ohnuma, Mio Kikuchi, Taichiro Ishige, Hironaga Kakoi, Kei-ichi Hirota, Yuji Takahashi, Shun-ichi Nagata

**Affiliations:** 1 Genetic Analysis Department, Laboratory of Racing Chemistry, 1731-2 Tsurutamachi, Utsunomiya 320-0851, Tochigi, Japan; 2 Equine Research Institute, Japan Racing Association, 1400-4 Shiba, Shimotsuke 329-0412, Tochigi, Japan

**Keywords:** gene doping, horseracing, INDEL, parentage test, SNV

## Abstract

Thoroughbreds are some of the most famous racehorses worldwide and are currently animals of high economic value. To understand genomic variability in Thoroughbreds, we identified genome-wide insertions and deletions (INDELs) and obtained their allele frequencies in this study. INDELs were obtained from whole-genome sequencing data of 101 Thoroughbred racehorses by mapping sequence reads to the horse reference genome. By integrating individual data, 1,453,349 and 113,047 INDELs were identified in the autosomal (1–31) and X chromosomes, respectively, while 18 INDELs were identified on the mitochondrial genome, totaling 1,566,414 INDELs. Of those, 779,457 loci (49.8%) were novel INDELs, while 786,957 loci (50.2%) were already registered in Ensembl. The sizes of diallelic INDELs ranged from −286 to +476, and the majority, 717,736 (52.14%) and 220,672 (16.03%), were 1-bp and 2-bp variants, respectively. Numerous INDELs were found to have lower frequencies of alternative (Alt) alleles. Many rare variants with low Alt allele frequencies (<0.5%) were also detected. In addition, 5955 loci were genotyped as having a minor allele frequency of 0.5 and being heterogeneous genotypes in all the horses. While short-read sequencing and its mapping to reference genome is a simple way of detecting variants, fake variants may be detected. Therefore, our data help to identify true variants in Thoroughbred horses. The INDEL database we constructed will provide useful information for genetic studies and industrial applications in Thoroughbred horses, including a gene-editing test for gene-doping control and a parentage test using INDELs for horse registration and identification.

## 1. Introduction

Thoroughbreds are some of the most famous racehorses worldwide. They had a founding population (of Arabian stallions and British mares) around the 18th century, and have been bred as a closed group for approximately 400 years [1]. In the current racing industry, over 80,000 Thoroughbred racehorses are born worldwide every year [2], and they are currently animals of high economic value.

The horse genome was sequenced and assembled in 2007 as EquCab2.0, in which 2.33-Gb draft sequences were published [3]. The latest version of the horse genome, EquCab3.0, was assembled as a total read length of 2,506,949,475 bp (1–31 and X: 2,409,143,234 bp, unplaced: 97,806,241 bp, Assembly: GCA_002863925.1), in 2019 [4]. By mapping sequence reads obtained from massive parallel sequencing to the reference genome sequences, genome-wide variants have been easily identified. Currently, whole-genome sequencing (WGS) of 88 horses (25 breeds) and 534 horses (46 breeds) has identified approximately 23.6 million and 29.0 million single nucleotide variants (SNVs), respectively [5,6]. In a Thoroughbred population of 101 unrelated horses, 12 million SNVs with their allelic frequencies were identified using WGS [7].

Insertions and deletions (INDELs) are variants consisting of different allele sizes (one or more nucleotides) in the genome. When INDELs occur in amino acid-coding regions, they are generally known to result in a loss of function. Although the existence of INDELs in coding regions is deleterious, their roles and functions in the population are not fully elucidated.

INDELs are highly abundant in human and animal genomes, and approximately 2.4 million and 2.1 million INDELs were identified from 88 and 534 horses, respectively [5,6]. In the Ensembl, 3,461,675 INDELs have been identified in the current horse assembly (https://ftp.ensembl.org/pub/release-108/variation/gvf/equus_caballus/, accessed on 2 February 2023). However, although several Thoroughbred horses were used for identification of genome-wide variants in the previous studies, the number, frequency, type, and size of INDELs in Thoroughbred horses have not been well elucidated.

Gene doping is a practice in horseracing that has been prohibited to maintain integrity [8]. One style of gene doping is to create genetically engineered animals; this has been carried out in many species, including horses [9,10,11]. The International Stud Book Committee (ISBC, https://www.internationalstudbook.com/, accessed on 2 February 2023) and the International Federation of Horseracing Authorities (IFHA, https://www.ifhaonline.org/, accessed on 2 February 2023) has prohibited the use of genetically engineered horses. In addition, horses born from genetically engineered animals are recognised as engineered horses. Editing (insertion and deletion) of coding genes causes loss of function. For instance, knocking out the *myostatin* gene, which is known as a negative regulator of muscle growth, may affect body composition and racing performance [12,13].

Recently, a gene-editing test was developed to detect artificially edited sequences using the clustered, regularly interspaced, short palindromic repeats/CRISPR-associated proteins (CRISPR/Cas) [14]. This test defined homologous insertions or deletions of novel variants as a type of artificial modification. Therefore, it is also necessary to identify INDELs in current Thoroughbred populations.

Microsatellites have been recently used for parentage testing in horse registration. While about 12–20 markers have been used for construction of a panel, because of their multiple alleles, they have the disadvantage of high mutation rates due to slippage errors. Therefore, construction of a new panel using SNVs or INDELs that have low mutation rates is expected. However, it is difficult to find many polymorphic SNPs and INDELs.

In our previous study [7], we presented only genome-wide SNV detection and their frequency data. This study focused on the detection of genome-wide INDELs and their frequency data. This study aimed to identify INDELs in a Thoroughbred population from 101 WGS data and to construct an INDEL database to provide a reference for genetic studies and industrial applications in horses, including pedigree registration and gene-doping control.

## 2. Materials and Methods

### 2.1. Whole-Genome Sequencing Data from 101 Thoroughbred Horses

FASTQ (DDBJ: SAMD00573909 to SAMD00574009) of WGS data (150-bp pair-end reads) from 101 Thoroughbred horses (58 males and 43 females) were used in this study (Table S1). The 101 horses were registered as Thoroughbred horses in Japan; some horses were born in other countries and were imported to Japan [7].

### 2.2. INDEL Calling and Filtering

Short INDELs were identified using the RESEQ pipeline (Amelieff Co., Minato, Tokyo, Japan). The pipeline was constructed using QCleaner (Amelieff Co.), Burrows–Wheeler Aligner (BWA, version 0.7.17) (https://bio-bwa.sourceforge.net/, accessed on 2 February 2023), Picard (version 2.13.2) (https://sourceforge.net/apps/mediawiki/picard/, accessed on 2 February 2023), GATK HaplotypeCaller (version 4.0.8.1) (https://software.broadinstitute.org/gatk/best-practices/, accessed on 2 February 2023), and SnpEff (version v4_0) (http://pcingola.github.io/SnpEff/download/, accessed on 2 February 2023). Quality control using QCleaner eliminates sequence reads for the following criteria: reads with a low-quality base (<20 Phred score), a quality value < 20 in 80% of their nucleotides, sequences of over five unknown nucleotides, only <32 bp length sequences, and those that are not mate pairs.

In brief, after qualification, reads obtained from WGS were aligned to the horse reference genome assembly EquCab3.0 (GenBank: GCA_002863925.1) using the BWA with default parameters to obtain a BAM file. Duplicate reads were removed using the Picard tool. GATK HaplotypeCaller detected insertions and deletions, and then filtered using the VariantFiltration program based on the following criteria: cluster WindowSize: 10; MQ0 ≥ 4 and ((MQ0/(1.0 × DP)) > 0.1), DP < 10, QUAL < 30.0, QUAL ≥ 30 and QUAL < 50, QD < 1.5, HRun > 5, SB > −0.1. Detected INDELs were annotated using SnpEff. Finally, all the annotated information was provided in variant call format (VCF) files. An Integrative Genomics Viewer (Broad Institute) was used for visualising mapping data using BAM files and variant data using VCF files.

### 2.3. Statistical Analyses of Identified INDELs

The chromosome, position, reference (Ref) allele, alternative (Alt) allele, gene name, HGVSp, annotation, and annotation impact were collected from the VCF files of 101 horses and then integrated by Vcf2sql (Amelieff Co.). The allele frequency of diallelic INDELs on autosomal (1 to 31) chromosomes was calculated from the integrated data by Vcf2sql. Statistical software R (https://www.r-project.org/, accessed on 2 February 2023) and its package (tidyverse package, version 1.3.2) were used for statistical analyses of identified INDELs.

INDEL density was calculated as the number of detected variants in each chromosome multiplied by a scale factor of 1000 to calculate the number of INDELs per 1000 bp.

Identified INDELs were compared with those registered in Ensembl Release 108 (https://ftp.ensembl.org/pub/release-108/variation/gvf/equus_caballus/, accessed on 2 February 2023) using statistical software R.

SNV data identified in the 101 horses were obtained from our previous study [7] and the following site (https://doi.org/10.17605/OSF.IO/PVNCY, accessed on 2 February 2023). To map genome-wide INDELs and SNVs on the horse ideogram, R Ideogram (https://cran.r-project.org/web/packages/RIdeogram/vignettes/RIdeogram.html, accessed on 2 February 2023) was used.

## 3. Results

### 3.1. Numbers of Detected INDELs

The mapped region (×10) and coverage of sequence reads were represented as the averages of 2,438,085,403 bp (2,384,904,267–2,557,836,663) and 36.8 coverages (29.5–54.2), respectively (Table S1). These data were considered sufficient for INDEL identification from the Thoroughbred genome because coverage of almost all horses was over 30, which is suitable coverage for identifying genome-wide variants [15]. The number of detected and filtered INDELs was identified with averages of 629,028 (532,978–673,567) and 585,680 (467,271–644,315), respectively (Table S1).

By integrating filtered INDELs, 1,453,349 and 113,047 loci were identified in the autosomal (1–31) and X chromosomes, respectively (Table 1 and Table S2). The majority of INDELs only had two alleles as ‘diallelic’ INDELs, while the others had three or more alleles as ‘multiallelic’ INDELs, which were tandem repeat sequences similar to microsatellites. Multiallelic INDELs were identified as 178,641 (12.29%) and 11,081 (9.80%) loci on the autosomal and X chromosomes, respectively (Table 1 and Table S2). Furthermore, 18 INDELs were detected by mapping to the mitochondrial (MT) genome (16,660 bp) (Table 1 and Table S2). In total, 1,566,414 INDELs were identified through the genome of the 101 Thoroughbred horses.

Of the 1,566,414 INDELs, 786,957 loci (50.2%) were already registered in the Ensembl (Release 108), while 779,457 loci (49.8%) were novel INDELs (Table S3). Since 3,461,675 INDELs are registered in the Ensembl, the INDELs identified in this study occupied 22.7%.

As described below (see Section 3.4), INDELs with a minor allele frequency (MAF) of 0.5 and heterozygous genotype in all horses were counted independently, because it was unclear whether they were true variants. Among diallelic loci, excluding a MAF of 0.5 and heterozygous genotype in all horses, 676,249 (53.3%) were novel INDELs and 592,504 (46.7%) were registered INDELs (Table S3). Among diallelic loci with MAF of 0.5 and heterozygous genotype in all horses, 3199 (53.7%) were novel INDELs and 2756 (46.3%) were registered INDELs (Table S3). Among multiallelic loci, 44,299 (23.3%) were novel INDELs and 145,425 (76.7%) were registered INDELs (Table S3).

As 1,566,396 INDELs were detected in the Thoroughbred population through chromosomes 1–31 and X, one INDEL was detected every 1538 bp (=2,409,143,234 bp as the total base pairs of all chromosomes/1,566,396 INDELs) on average.

**Figure 1:**
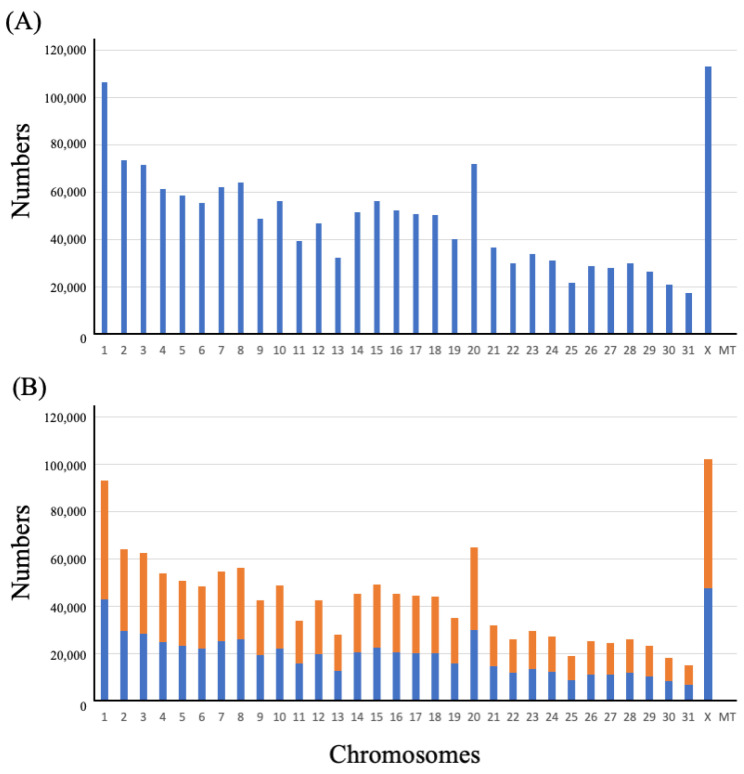
Distribution of insertions and deletions (INDELs) detected in 101 Thoroughbred horses. (**A**) Distribution of all INDELs identified on chromosomes 1–31, X, and MT; and (**B**) Distribution of insertions and deletions of diallelic INDELs on chromosomes 1–31, X, and MT (blue: insertions; orange: deletions).

Figure 1A shows the number of INDELs detected on each chromosome. Chromosomes 12 (on average 1.26 in 1000-bp as INDEL density) and 20 (1.10) showed a frequency of 1 or more in 1000 bp (Table S2). Except for these chromosomes, there were <1.0 in 1000 bp (0.54 in chromosome 14 to 0.88 in chromosome X, Table S2).

### 3.2. Sizes of Detected INDELs

**Figure 2:**
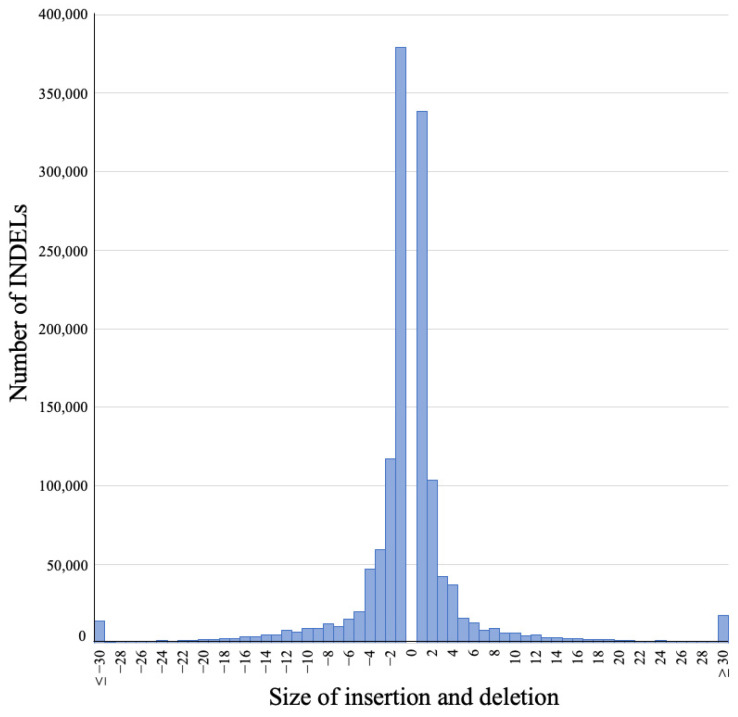
Distribution of insertion and deletion (INDEL) size detected in 101 Thoroughbred horses. The size of INDELs was calculated as Alt allele length–Ref allele length.

In 1,376,674 diallelic INDELs in autosomal and X chromosomes, 742,723 (54.0%) and 633,951 (46.0%) loci were detected as deletions and insertions, respectively (Figure 1B). The sizes of the detected INDELs (calculated as Alt allele length−Ref allele length) were in the range of −286–+476, and 1-bp, 2-bp, 3-bp, and 4-bp INDELs accounted for the majority, with 717,736 (52.14%), 220,672 (16.03%), 101,756 (7.39%), and 83,780 (6.09%), respectively (Figure 2).

### 3.3. Allelic Frequency Distribution of INDELs Detected

**Figure 3:**
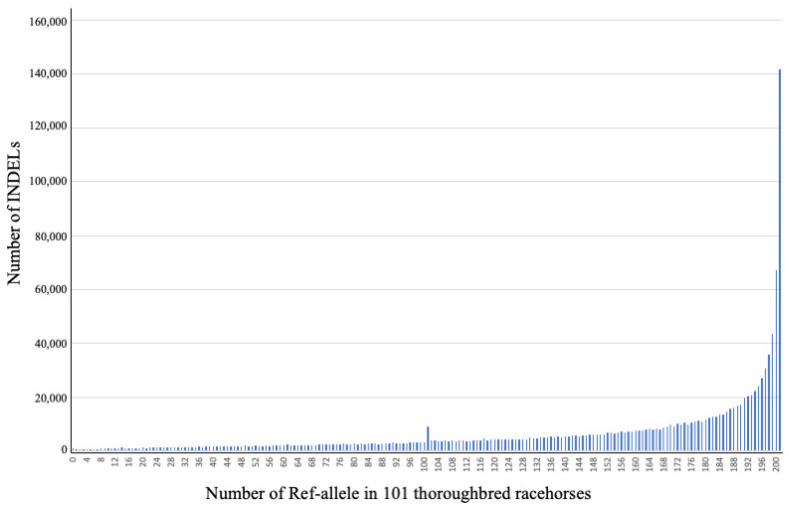
Allelic distribution of 1,274,708 diallelic insertions and deletions (INDELs) identified on autosomes (chromosomes 1–31) in 101 Thoroughbred racehorses. The horizontal axis indicates the number (frequency) of Ref alleles in the population (202 alleles in total), meaning that the right edge shows a MAF of 0.00495 for Alt allele, the centre shows a MAF of 0.5, and the left edge shows a MAF of 0 (Alt/Alt genotypes in all horses).

The allelic frequency distributions of 1,274,708 diallelic INDELs identified on autosomes were investigated in 101 Thoroughbred racehorses. The horizontal axis of the figure indicates the number (frequency) of Ref alleles in the population (202 alleles in total), meaning that the right edge shows a MAF of 0.00495 (<0.5%) for Alt allele, the centre shows MAF of 0.5, and the left edge shows a MAF of 0 (Alt/Alt genotypes in all horses). Many INDELs were detected as having a smaller frequency of Alt alleles. In particular, many rare variants with a low Alt allele frequency (<1%) in the population were detected (Figure 3).

In the 1,274,708 diallelic INDELs, 141,686 loci had a MAF of 0.00495 for Alt alleles (see the right edge of Figure 3), whereas 881 loci showed Alt allele homozygotes for all individuals in the population (see the left edge of Figure 3). In addition, a blip was observed at 101 Ref alleles/101 Alt alleles (see the centre of Figure 3), which corresponds to a MAF of 0.5. Interestingly, although 9228 INDELs on autosomal chromosomes (1–31) had a MAF of 0.5, 5955 (64.5%) were of the heterozygous genotype in all horses.

In the diallelic INDELs identified on chromosomes 1 to 31, excluding variants with a MAF of 0.5 and all heterozygotes in all horses, novel INDELs that were not registered in the Ensembl (Release 108) were mainly distributed in fewer Alt allele counts in 101 Thoroughbred racehorses (Table S4); this is similar to the frequency distribution in Figure 3, meaning that many novel INDELs were detected as rare variants. Interestingly, novel 317 loci were identified as all Alt allele homozygotes in the 101 horses, meaning that all the individuals analysed did not have reference variants for the 317 loci (Table S4).

### 3.4. Detection of Genomic Regions Having MAF of 0.5 and All Heterozygous

**Figure 4:**
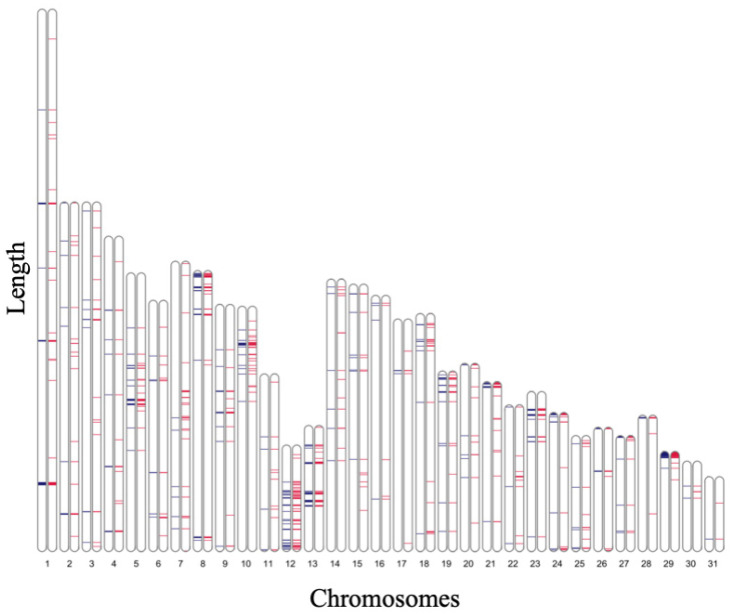
Locations of insertions and deletions (INDELs) and single-nucleotide variants (SNVs) with a MAF of 0.5 and heterozygous genotypes in all horses. Blue: INDELs detected in this study; pink: SNVs detected in our previous study. Many regions are matched between the loci of SNVs and INDELs. The pericentromeric region (position: 1–2,261,991, approximately 2.26 Mb) of ECA29 was densely distributed.

The genomic regions of the 5955 INDELs with a MAF of 0.5 and heterozygous genotype in all horses were visualized on horse chromosomes (Figure 4, blue). In our previous study [7], 58,582 loci on chromosomes 1–31 were identified as SNVs with a MAF of 0.5 and heterozygous genotypes in all horses. These SNVs were also visualized on the horse chromosomes (Figure 4, pink). These genomic regions were densely distributed with SNVs and INDELs, and the regions were similar.

Notably, 1137 of these INDELs were in the pericentromeric region (position: 1–2,261,991) of ECA29. A similar trend was observed in SNVs with a MAF of 0.5 and heterozygous genotype in all horses (chromosome 29: 1–2,293,071). In addition, the other INDELs and SNVs with a MAF of 0.5 and heterozygous genotypes in all horses were widely distributed over short lengths (Figure 4). INDELs with a MAF of 0.5 and heterozygous genotype in all horses presented more densely in chromosome 12 compared to other chromosomes. These regions showed high coverage of mapped reads upon observation with the Integrative Genomics Viewer. Complementing the distribution patterns of SNVs and INDELs would enable the elucidation of more detailed genomic structures.

### 3.5. Characterisation of INDELs Detected

The number of INDELs detected in the functional region of the genome was also investigated (Table 2). Although most INDELs were in the intergenic and intron regions (Table S5), 12,432 loci were found in exons (including non-coding RNA). Of these, 4529, 1871, 5709, and 323 were frameshifts, non-frameshifts, long non-coding RNAs and pseudogenes, respectively. As known non-frameshift INDELs in horses, an INDEL of the agouti signalling protein (*ASIP*) gene was detected with a reference allele frequency of 0.248, while a short interspersed nuclear element (SINE) insertion was not detected in the myostatin (*MSTN*) gene region, based on our detection criteria.

**Figure 5:**
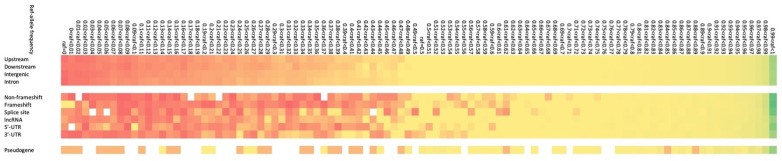
Distribution density of insertions and deletions (INDELs) in genomic functional regions. The distribution density (0.640%: yellow) of frameshift INDELs was higher than that of non-frameshift INDELs (0.160%: red) in the 0% Ref allele frequency (left side of Figure 5). Vertical line: upstream, downstream, intergenic, intron, non-frameshift, frameshift, splice site, long non-coding RNA, 5′-UTR, and 3′-UTR. Horizontal line: reference allele frequency, left side: low frequency, right side: high frequency. Red, yellow, and green show low, middle, and high densities, respectively.

Notably, 29 INDELs located in exons of 23 genes all had alternative alleles, causing frameshifts in the 101 horses (Tables S6 and S7). The distribution density (0.640%) of frameshift INDELs was higher than that of non-frameshift INDELs (0.160%) in the 0% Ref allele frequency (left side of Figure 5). While 5 genes were not annotated (ENSECAG00000020750, ENSECAG00000022530, ENSECAG00000026851, ENSECAG00000030750, and ENSECAG00000033068), 18 genes were annotated as follows; ankyrin repeat domain 9 (*ANKRD9*), Rho GTPase-activating protein 45 (*ARHGAP45*), cache domain containing 1 (*CACHD1*), cardiotrophin 1 (*CTF1*), EMAP-like 4 (*EML4*), fibrosin-like 1 (*FBRSL1*), HDGF-like 2 (*HDGFL2*), junctophilin 2 (*JPH2*), mitochondrial ribosomal protein L15 (*MRPL15*), nuclear receptor corepressor 2 (*NCOR2*), SH3 and multiple ankyrin repeat domains 3 (*SHANK3*), serine/threonine kinase 11 (*STK11*), trinucleotide repeat-containing 18 (*TNRC18*), UPF1 RNA helicase and ATPase (*UPF1*), zinc finger protein 212 (*ZNF212*), zinc finger protein 282 (*ZNF282*), zinc finger protein 516 (*ZNF516*), and zinc finger protein 853 (*ZNF853*).

## 4. Discussion

In this study, 1,566,414 loci were identified as INDELs on autosomal and X chromosomes and mitochondrial genomes. It was confirmed that 779,457 loci (49.8%) were novel INDELs by comparing them with the Ensembl (Release 108). Our current and previous studies [7,16] identified 12 million SNVs, 1.56 million short INDELs, and 62 processed pseudogenes in 101 Thoroughbred horses. Although Thoroughbreds were bred as a closed population from a small founder population, it was demonstrated that current Thoroughbred populations have diverse genome structures.

A recent study estimated a rate of 2.94 INDELs (1–20 bp) and 0.16 structural variants (>20 bp) per generation based on the WGS of 250 human families [17]. In this study, 96.36% of diallelic INDELs were in the size range of 1–20 bp. Therefore, although many rare INDELs were detected in the present study, a greater number of de novo INDELs may be identified by analysing Thoroughbreds other than the 101 horses analysed in this study. However, for de novo INDELs to remain in Thoroughbred populations, they must occur in stallions or broodmares. As few horses are used as breeding stallions or broodmares, many de novo INDELs in other horses will be lost without inheritance. In this study, many INDELs were detected as rare variants (e.g., INDELs with 1 Alt allele count in the 101 horses). These INDELs are variants that may not have remained in the population.

Interestingly, 5955 loci were genotyped with a MAF of 0.5 and heterogeneous genotypes in all the horses. Such genotype distribution is unlikely in a randomly collected population. Because these variants existed in continuous positions with high mapping coverage, fake variants may be detected by mapping of similar sequence reads in multiple regions. While short-read sequencing and its mapping to the reference genome is an easy way to detect variants, fake variants may be detected. Therefore, our data help to identify true variants in Thoroughbreds. As 2756 loci were registered in Ensembl (Release 108), further validation, such as designing their primers and probes, may need to use them as genetic markers.

High INDEL density was observed on chromosomes 12 and 20. Previous studies observed a similar trend on the same chromosomes for SNVs and nonsynonymous substitutions [7,16]. Although the exact reason for this is unknown, one reason is thought to be the existence of metabolic and sensory perception regions on chromosome 12, and immune response and antigen-processing regions on chromosome 20 [18,19,20]. These genes have several pseudogenes that lose their function because of substitutions, insertions, and deletions in the gene regions. In addition, INDELs were densely populated on chromosome 12, perhaps because of the effect of duplicated regions.

INDELs in coding regions may be frameshift or non-frameshift variants. In this study, the same variant (C/CAGCAGAAAAGA) as a non-frameshift INDEL in the *ASIP* gene was observed, and its genotypes were associated with coat colour [21,22]. In addition, SINE insertion in the *MSTN* gene is associated with optimum race distance and muscle mass in Thoroughbred racehorses [23,24]. However, the SINE insertion was not detected in our analysis pipeline (see Materials and Methods), while reads mapped to the promoter region in *MSTN* had partial sequences soft-clipped by confirmation using the Integrative Genomics Viewer. Although INDELs over 400 bp were detected in this study; the SINEs in the *MSTN* region were not recognised as INDELs by our detection method, because similar sequences were widely distributed in the horse genome.

Frameshift INDELs are generally deleterious and contribute to disease susceptibility, as evidenced by human genomics research that has identified many INDELs related to genetic diseases including cancer [25]. Notably, 29 INDELs detected in exons of 23 genes had Alt alleles causing frameshifts in the 101 horses, indicating that all genotypes were frameshifted in the population. Interestingly, four of them were family genes of zinc finger protein, ZNF212, ZNF282, ZNF516, and ZNF853. Zinc finger proteins are generally involved in gene regulation and development thorough binding to DNA or RNA. Although no functional commonality was observed in the other detected genes, some of them seem to involve in intracellular signalling, such as FBRSL1, NCOR2, and UPF1. Although these genes were important for cellular functions, they may be complemented by different signalling pathways even if the genes did not work well by frameshifting. It is expected that genes containing INDELs will acquire special functions by frameshifting, and that the genome structure of Twilight (the reference genome) will be unique. However, mistakes in genome assembly, gene annotation, and INDEL calling cannot be ruled out. Further detailed studies are needed because INDELs identified in exons may be related to positive selection, genetic diseases, and/or diverse functions in Thoroughbreds.

In human forensic sciences, INDELs have been noted as markers for individual identification testing [26,27] because they are diallelic, small in size, and widely distributed throughout the genome. Furthermore, INDELs have the advantage of lower mutation rates than SNPs and short tandem repeats (STRs) [28]. In the present study, we propose 97,443 INDELs (chromosome: autosomes, allele number: diallelic, size: −2 to −4 and +2 to +4, allele frequency: 0.25 to 0.75, excluding INDELs with all heterozygous genotypes) as marker candidates to construct a panel of horse parentage testing. Registration of Thoroughbred racehorses requires parentage testing using STRs recommended by the International Society for Animal Genetics [29,30]. While parentage testing using SNPs is being developed [31,32], the INDEL panel may also serve as a complementary panel.

Gene doping in the horseracing industry is defined as administration of exogenous genes or therapeutic oligonucleotides to postnatal animals, and the creation of genetically modified animals. These have been prohibited by the IFHA and the ISBC. While the former can be target-specifically detected by quantitative PCR using a hydrolysis probe or sequencing using matrix-assisted laser desorption/ionisation time-of-flight mass spectrometry [33,34], the latter has been extremely difficult to detect. One of the reasons for this is that we do not fully understand the genomic diversity of Thoroughbred populations. Therefore, the results of our study we consider to contribute to gene-doping control. Recently, we developed a gene-editing test to detect these racehorses [14]. This test uses the following criterion to identify artificial editing: the presence of homologues of Alt-type INDELs that were not shown in current Thoroughbred populations. Therefore, as this study analysed and validated the types, sizes, locations, and frequencies of INDELs in the current Thoroughbred population, the results will contribute to the gene-editing test. Interestingly, this study identified many rare INDELs which are of low frequency in the Thoroughbred population. While rare INDELs may be naturally occurring as de novo mutations, the presence of rare INDELs may complicate positive or negative determination in the gene-editing test. If de novo mutations occur in stallions, the mutations are inherited by many offspring. Therefore, the variant database constructed in this study should be regularly updated for accurate gene-editing testing. However, our data may include up to several thousand false-positive variants calls because of current mapping and variant-calling algorithms. Therefore, developing improved variant-calling algorithms is a future research priority and is required for industrial applications such as precise gene-doping control.

The INDEL database we have constructed will provide useful information for genetic studies and industrial applications in Thoroughbred horses, including a gene-editing test for gene-doping control and a parentage test using INDELs for horse registration and identification.

## Figures and Tables

**Table 1:** The number of INDELs detected in 101 Thoroughbred racehorses.

INDEL Category	Chromosomes 1 to 31	Chromosome X	Mitochondria
All INDELs	1,453,349	113,047	18
Diallelic INDELs	1,274,708	101,966	16
Multiallelic INDELs	178,641	11,081	2

**Table 2:** Characterisation of insertions and deletions detected in 101 Thoroughbred racehorses.

Region	Chromosomes 1 to 31	Chromosome X	Mitochondria
Upstream	101,434	5633	16
5′-UTR	2071	85	0
Exon	12,432	870	7
Intron	508,310	33,247	0
3′-UTR	3258	227	0
Downstream	96,718	5989	16
Intergenic	754,952	67,934	3

UTR: untranslated region.

## Data Availability

The data supporting the findings of this study can be accessed through the Open Science Framework (https://OSF.IO/QN45H/, accessed on 2 February 2023). FASTQ data used in this study are available from the DDBJ (BioProject: PRJDB15140, BioSample: SAMD00573909 to SAMD00574009, DRA015615).

## Supplementary Materials

The following supporting information can be downloaded at: https://www.mdpi.com/article/10.3390/genes14030638/s1, Table S1: Summary of mapping and INDEL calling; Table S2: Number of INDELs detected at individual chromosomes in 101 Thoroughbred racehorses; Table S3: The number of INDELs identified in this study; Table S4: Distribution of novel diallelic INDELs excluding MAF of 0.5 and heterozygous genotype in all horses; Table S5: Characterisation of INDELs detected at individual chromosomes in 101 Thoroughbred racehorses; Table S6: INDEL distribution based on allele frequency; Table S7: Genes that are homozygous for alternative alleles and are the cause of frameshifts in all 101 horses.
